# Recent Developments of Silk-Based Scaffolds for Tissue Engineering and Regenerative Medicine Applications: A Special Focus on the Advancement of 3D Printing

**DOI:** 10.3390/biomimetics8010016

**Published:** 2023-01-02

**Authors:** Asma Musfira Shabbirahmed, Rajkumar Sekar, Levin Anbu Gomez, Medidi Raja Sekhar, Samson Prince Hiruthyaswamy, Nagaraj Basavegowda, Prathap Somu

**Affiliations:** 1 Department of Biotechnology, School of Agriculture and Biosciences, Karunya Institute of Technology and Sciences (Deemed-to-be University), Karunya Nagar, Coimbatore 641 114, Tamil Nadu, India; 2 Department of Chemistry, Karpaga Vinayaga College of Engineering and Technology, GST Road, Chinna Kolambakkam, Chengalpattu 603308, Tamil Nadu, India; 3 Department of Chemistry, College of Natural Sciences, Kebri Dehar University, Korahe Zone, Somali Region, Kebri Dehar 3060, Ethiopia; 4 Department of Biotechnology, Rathinam Technical Campus, Eachanari, Coimbatore, Tamil Nadu 641021, India; 5 Department of Biotechnology, Yeungnam University, Gyeongsan 38541, Republic of Korea; 6 Department of Bioengineering, Institute of Biotechnology, Saveetha School of Engineering, Saveetha Institute of Medical and Technical Sciences (Deemed to be University), Chennai 600124, Tamil Nadu, India

**Keywords:** fibroin, 3D printing, regenerative medicine, tissue engineering, biomaterials, scaffolds

## Abstract

Regenerative medicine has received potential attention around the globe, with improving cell performances, one of the necessary ideas for the advancements of regenerative medicine. It is crucial to enhance cell performances in the physiological system for drug release studies because the variation in cell environments between in vitro and in vivo develops a loop in drug estimation. On the other hand, tissue engineering is a potential path to integrate cells with scaffold biomaterials and produce growth factors to regenerate organs. Scaffold biomaterials are a prototype for tissue production and perform vital functions in tissue engineering. Silk fibroin is a natural fibrous polymer with significant usage in regenerative medicine because of the growing interest in leftovers for silk biomaterials in tissue engineering. Among various natural biopolymer-based biomaterials, silk fibroin-based biomaterials have attracted significant attention due to their outstanding mechanical properties, biocompatibility, hemocompatibility, and biodegradability for regenerative medicine and scaffold applications. This review article focused on highlighting the recent advancements of 3D printing in silk fibroin scaffold technologies for regenerative medicine and tissue engineering.

## 1. Introduction

According to the World Health Organization, only 10% of the world’s need for tissue and organs is being fulfilled, making it a severe public health issue. Additionally, the long-term success of organ transplantation is unknown as recipients must take lifelong immunosuppressive treatment regimens, increasing the risk of fatal infections, and because half of the transplants fail after ten years [1]. Other difficulties with tissue/organ transplantation include ethical permission, persuading family members to give tissue/organs, and the fact that many hospitals, particularly in middle- and low-income countries, lack the resources to maintain the organs/tissue of brain-dead patients [2]. In addition to tissue/organ transplantation, a significant problem faced by biomedical research organizations is figuring out the cellular and molecular causes of human disease to provide novel methods for therapeutic intervention, prevention, or diagnosis [3]. To gain valuable insights into disease mechanisms, medication testing, and safety evaluations, scientists have used two-dimensional cultures, animal models, or cadavers for decades. However, human translation’s reliability, relevance, and repeatability are in doubt [4]. Developing in vitro tissue/organ equivalents and bioartificial tissue/organs that maximize their availability while reducing immune reactions to save lives have been the two main drivers of recent breakthroughs in tissue engineering and regenerative medicine (TERM) [5]. The unit’s manual traditional tissue engineering process was time-consuming and technically challenging [6]. Another difficulty with standard TERM approaches is reproducing organ complexity and revascularizing implanted tissue/organ at the human scale [7]. Research on the creation of preclinical models and bioartificial organs has been accelerated by the development of automation technologies and innovative biomaterial compositions [8].

The natural protein known as silk fibroin (SF) has excellent mechanical qualities, good cell compatibility, predictable degradation, and flexible processing in various material formats [9]. In several tissue-engineering applications, including the regeneration of urethras, skin, tendons, and bones, the potential of SF nanofibers has been studied. One of the main approaches in treating tissue or organ failure involves regenerating tissues employing cells, scaffolds, and the proper growth hormones. Fibroin, a silk protein, can function well as a biomaterial in various therapies. Numerous creatures, including spiders, silkworms, scorpions, mites, and flies, are used to produce silk strands. One of these is silk from silkworms, which can be used to create biomedical devices. It is manufactured in large quantities for the textile industry and has suitable mechanical qualities and strong biocompatibility. Silk fibroin is a desirable material for tissue engineering because of its unique blend of strength, flexibility, and compatibility with mammalian cells [10]. 

A natural biopolymer that is widely accessible and has an antiquity of use as sutures in the human body is silk fibroin from silkworms. Currently, silk sutures are used to cure skin wounds and operate on the lips, eyes, and mouth [11]. Due to an increasing understanding of its fabrication and features, including physical strength, elasticity, biocompatibility, and regulated biodegradability, silk fibroin is being used more and more in different fields of biomedical science. Silk fibroin’s unique characteristics make it a valuable material for tissue engineering [10].

Recent research also assesses silk as a component of optical systems for diagnostics and real-time functional and physiological recording on flexible electronic devices [12]. Silk has excessive surface smoothness, aqueous processing, and good (about 95%) optical transparency throughout the visible range, all of which promote its use in optic and photonic biosensors [13]. These silk-based devices offer the capability and sensitivity required for sophisticated applications and are implantable. Several reviews have been written on the creation, composition, and use of silk-based biomaterials [14]. Table 1 stands as evidence of the fact that innovations in the area of silk fibroin-mediated biomedical devices hold enormous commercial prospects.

A more thorough study is now necessary in light of the expanding applications of silk in novel tissue engineering fields and our improving understanding of the properties of silk constructions [15].

This review paper examines recent advancements in silk fibroin-based tissue regeneration research and assesses their potential for future growth in therapeutic-related applications. An overview of silk protein is covered at the start of the review. The structure and morphologies of the silk protein fibroin are included as they are crucial to the use of silk biomaterials in tissue engineering. Following a discussion of the various silk-based platforms, a description of how they are used in tissue regeneration is given. 

## 2. Sources, Structure, and Chemistry of Silks Derived from the Silkworms

Arthropods that produce silk (such as spiders, bees, silkworms, mites, and scorpions) have glands that contain silk proteins, which are spun into fibers during their metamorphosis. Silk from silkworms is a well-known material widely used in the textile industry. However, the industrial production of spider silk is constrained by spiders’ cannibalistic behavior [16]. The fiber of a silk cocoon can be as long as 600 to 1500 m, whereas the fiber of a spider web can be 137 m long [17]. Spider silks have a diversified structure as well. As a result, biomaterials produced from silk are frequently created from the silk of the silkworm. The silk manufactured by Bombyxmori, an affiliate of the Bombycidae family, is unique. Mulberry silk is another name for B. *mori* silk. Saturniidae is another silk-producing family, and its product is referred to as non-mulberry silk [18].

Silk has many significant benefits compared to other protein-based biomaterials produced from allogeneic or xenogeneic tissues. As a result, those materials have a substantial risk of infection [19]. The expensive processing of such materials results from severe protein separation and purifying procedures. In comparison, silk is a well-recognized textile fiber, and each year, the production and processing of silk totals close to 1000 metric tonnes. A straightforward alkali or enzyme-based degumming technique is frequently used to purify silk fibers, producing the raw material for sericin-free silk-based biomaterials [20]. Because of the large-scale infrastructure for processing that exists within the traditional silk textile industry, using silk for biomedical applications is also financially advantageous [10].

A large molecular weight (200–350 kDa or more) and bulky repeated modular hydrophobic domains with tiny hydrophilic groups in between characterize silk [21]. The silk fibroin’s N and C termini are very reticent. Due to the presence of disulfide linkage, the heavy (H) and light (L) chains are connected in B. *mori*’s silk fibroin. Moreover, 25 kDa of glycoprotein (P25) is also conjugated with these chains in a non-covalent manner [22]. The hydrophobic domains of H chains can form anti-parallel sheets and contain Gly-X (X being Ala, Ser, Thr, or Val) repeats. The L-chain has a hydrophilic and somewhat stretchy character. P25 protein is crucial in preserving the complex’s integrity [23]. In mulberry silk, H-fibroin, L-fibroin, and P25 are put together in a 6:6:1 ratio [24]. P25 and the light (L) chain are absent in non-mulberry silks. Instead, they comprise separate proteins (160 kDa) that combine to create H-chain homodimers (330 k Da) [25]. Moreover, they have larger ratios of amino acids [26]. Due to these changes, mulberry and non-mulberry silks exhibit significantly different mechanical characteristics, bioactivity, and degradation behavior [27]. 

Many of silk fibroin’s biomaterial features depend on its secondary structure and hierarchical organization in addition to its primary organization. The repeating amino acid sequence that composes the silk polymeric chains’ hydrophobic domains formed into nanocrystals (β-sheet). The bulky and polar side chains that comprise the hydrophilic linkages between these hydrophobic domains compose the amorphous portion of the secondary structure [28]. Silk has flexibility because of the random coil chain conformation found in amorphous blocks [29]. The specific regulation of size, distribution, number, 3D arrangement, and coordination at the nanoscale level is crucial in determining the mechanical properties of any specific silk. However, microstructural flaws such as vacuoles, microvoids, and nanocrystals help silk retain its exceptional mechanical capabilities [30]. A hierarchical supramolecular arrangement is also visible in silk fibers in addition to the secondary structure. Microfilament bundles (0.5–2 m) compose the spider and silkworm silks, and each contains nanocrystals and semi-crystalline domains [10]. All silkworm silk fibers adhere to the same hierarchical structural arrangements, notwithstanding some differences between silk kinds in the leading group and structural characteristics at the nanometer scale [31].

## 3. Features of Silk Fibroin as a Biomaterial 

For tissue engineering applications, silk fiber has the best mechanical properties among all natural biopolymers. Other notable characteristics include biocompatibility, water-based processing, biodegradability, and readily accessible chemical groups for functional modification. The following sections provide more details about these characteristics.

### 3.1. Physical Properties 

Silk provides a desirable blend of modulus, breaking strength, and elongation, which enhances its ductility and toughness. Kevlar, a benchmark in high-performance fiber technology, is weaker than silk fibers [32]. Compared to steel, silk has a strength-to-density ratio of up to ten times higher [33]. Particularly, spider silk fibers have remarkable extensibility and clearly show stress acclimatization tendency [34]. This stress-maintaining property is essentially required for energy-absorbing biomaterials. The form and strain hardening of wild silkworm threads’ stress–strain curves are comparable to those of spider or dragline silks [35]. Undoubtedly, silks are widely applicable as a biomaterial to generate scaffolds for tissue engineering due to the outstanding mechanical strength of silk fibers. Existing silk-based biomaterials do not fully explore the mechanical properties that can be derived from using multiple types of silk. When a biomaterial implant fails, it usually does so because it lacks the mechanical qualities required for the application or because the stress concentration at the implant–tissue interface is not acceptable [36]

Most silk materials created from silk fibroin solution are fragile, despite the original silk fibers’ remarkable mechanical characteristics. In comparison to innate fibers, which have a ductile strength of roughly 0.5–0.6 GPa and elongation at a break of 10–40% [37], silk film, for instance, has a dry tensile strength of 0.02 GPa and an elongation at break of less than 2%. This discrepancy between the regenerated materials and the native fibers might be related to the nonexistence of an adequate hierarchical and secondary structure [38]. Recent research demonstrates that modifying the structure during regeneration can considerably increase the sturdiness of remodeled silk goods to that of natural fibers or even advanced [39]. Such studies broadly apply to regenerated fibers and are still in the perception stage. It is necessary to make more efforts to increase the regenerated silk materials’ tensile and elongation strengths. Hence, tuning the mechanical strength for the required level based on tissue construction leads to extending the application of silk fibers in the area of tissue engineering. 

### 3.2. Biodegradability

The biodegradation studies of silk involve the weight reduction, morphological destructuring, and in vitro study of the degraded components; the biodegradation of silk is investigated. Similarly, degradation is examined in animal models using histological examinations, fluorescence staining, and other biochemical assays to examine structural integrity and assess the mechanical strength of scaffolds after embedding for a predetermined period. The regenerated silk fibroin scaffold shows faster degradation than the normal silk fiber scaffold. The secondary structure of silk created during the manufacture of regenerated silk materials affects the degradation rate [40]. 

The breakdown of silk materials is frequently discussed in terms of biodegradability. Biodegradability is the ability of an implanted polymer to break down into pieces that can move away from the site by fluid transfer but are not necessarily removed from the body [41]. Contrarily, biosorption completely removes the original foreign substance through filtering or metabolizing the bioproducts that have been broken down [42]. Their research shows that after being implanted in an in vivo model, aqueous-mediated 3D silk scaffolds started to fall apart after a few weeks and vanished entirely after a year. Protease XIV from Streptomyces griseus and α-chymotrypsin from the bovine pancreas are the most often utilized model enzymes for silk in in vitro models, respectively [43]. Gamma radiation can also be used to control the rate of enzyme breakdown [44]. It has been demonstrated that cells in vitro can also mediate the degradation of silk systems. Metalloproteinases (MMPs) and integrin expression by osteoblast and osteoclast cells could degrade silk films [45]. These positive findings are because the native extracellular matrix undergoes continuous in vivo remodeling via synchronized MMP-mediated proteolytic breakdown and matrix regeneration [46]. The biodegradability of various silk materials and architectures can be compared using in vitro research. 

In some areas of biodegradation, silk is superior to other biomaterials. Although degradation products for synthetic biomaterials such as polyglycolide and polylactides that have regulatory authority approval are resorbed by metabolic pathways, also generating by-products is a cause for worry. Such problems are not related to silk. Additionally, these synthetic materials may experience very early mechanical property degradation [47]. However, many silk systems’ ability to maintain strength over a long period might benefit silk, particularly for engineered tissue scaffolds, where gradual degradability, as well as heavy weight-bearing capability, are essential. Based on these benefits, further research is needed to fully understand how silk degrades and clears, stimulating silk’s development as a significant biodegradable.

### 3.3. Biological Competency

Silk is a material known to be biocompatible due to the lengthy history of the accomplishment of silk sutures [48,49]. However, silk proteins are not of mammalian origin and could cause adverse immunological events like other non-autologous biomaterials that trigger a foreign body response. Sericin, a protein resembling silk gum, is thought to cause some delayed hypersensitivity incidents of silk sutures in scarce circumstances [50]. Additional research using isolated sericin from silk and sericin-based biomaterials has not demonstrated that sericin is the cause of adverse effects [51]. To pinpoint the origin of any cytotoxic non-fibroin rudiments in silk and create an effective diagnostic method, thorough investigations are required.

K-fibroin bioconjugates have demonstrated a well-tolerated response when used to treat musculoskeletal diseases [52]. Although some normal phagocyte and lymphocyte buildup is seen, there is a lack of symptoms of sepsis with the hypodermic grafting of electrospun fiber mats in rats after 8 weeks. Additionally, under a microscope, hematoxylin and eosin-stained tissues revealed relatively minor inflammation [52,53]. After one year, a subcutaneously implanted silk 3D construct in vivo model produced an inadequate immune response [54].

Overall, these investigations show that appropriately sterilized and degummed silk products have acceptable biological competency and can be correlated with other widely used biomaterials, such as collagen and polylactic acid [55]. A few silk-based materials have acquired regulatory agreements for enlarged biomaterial devices for reconstructive and plastic surgeries due to in-depth studies conducted in recent years. For instance, testing for biocompatibility according to ISO 10993 under sound laboratory principles (GLP) reveals that silk-based surgical mesh Seri Fascia satisfies the standards [56].

Although the results are encouraging, there are still some uncertainties regarding the long-term safety of silk biomaterials in the human body. First, depending on how quickly the wound heals, silk sutures only stay in the body for a short while before being removed. The longstanding reactions of the inborn and acquired immune system built on the position of the implant area and type of construct utilizing suitable in vivo models demand additional research because silk products for tissue engineering require sustained contact with tissues [57].

Secondly, depending on their size and morphology, the degradation products of silk biomaterials may raise immunological reactivity issues [58]. It is acknowledged that producing particle debris, which could arouse the immune system, is one of the main reasons any biomaterial implant could fail. According to a report, silk fiber fractions can accelerate phagocytosis and produce a small amount of pro-inflammatory cytokines [54]. Similar results are obtained when the C-terminal of A. *pernyi* silk is digested with -chymotrypsin, showing reduced cytocompatibility and weak cellular attachment. Their finding indicates that B. *mori* solution may make it easier for amyloid to build up, leading to tissue deterioration. Therefore, long-term studies on deteriorated products are required to completely allay any worries about using silk-based constructs in biomedical implantation [59].

### 3.4. Modifying the Qualities of Silk by Altering the Structure

During spinning or regeneration, silk’s structure can be appropriately adjusted to produce various secondary structures that can be used to alter the qualities of the material. For instance, the forceful extrusion of silk gland protein via silkworm spinnerets results in a fiber microstructure that is appropriately changed and has a noticeably high level of fiber toughness [60]. If these choices are tailored for different silk-mediated biomaterials, they may have the benefit of identical weight-holding capabilities of the targeted tissues. To make silk products insoluble, water annealing is also utilized [61]. Compared to films treated with methanol, water-annealed films are more flexible and deteriorate more quickly [62]. There are fantastic chances to modify the structure and characteristics of materials made from regenerated silk using the protic ionic liquid system [63]. In addition, features including biodegradation, cell interaction, and drug release kinetics may be impacted by process-induced variations in structure and surface topography. For instance, the crystallinity of the silk films is programmed using a temperature-controlled water vapor annealing (TCWVA) process to adjust their thermal, mechanical, and biodegradation properties [64]. Likewise, structural modifications slowing down proteolytic degradation can lower the drug release kinetics from a silk system [65]. These situations show windows of opportunity, but further research is required to grasp structure-property linkages and regulate material attributes fully. The effectiveness of silk as a natural biopolymer for tissue regeneration will depend on this regulation.

## 4. Structural Diversification of Silk Biomaterials 

The initial stage of processing silk fibers is degumming, i.e., separating the adhesive protein sericin. Silk solution is created by dissolving degummed silk fiber to generate various other material types. When dissolving fibers is challenging, fibroin can occasionally be isolated straightly from the silkworm glands using the right buffer solution. Then, using liquid-to-solid phase transfer, several solid forms of silk are obtained from the silk solution. For tissue engineering, materials created from natural fibers, as well as those created from silk solution, are used.

### 4.1. Innate Silk Structures

In tissue regeneration, degummed silk strands can be twisted to create various twisted structures such as ropes, cables, and braided and textured yarns [66]. Additionally, by partly dissolving cocoons to act as a cell-supporting prototype, non-woven constructions can be created, with the grouping of the filaments in the cocoon being conserved to preserve the porosity construct of the mat [67]. Constructing a knitted silk framework to support 3D porous engineered tissue is an alternative method of directly employing silk filaments in tissue engineering. By adding reinforcement, scaffolds’ mechanical qualities are enhanced for use in the ligament, a load-bearing structure in tissue engineering.

### 4.2. Silk Films

The aqueous, acidic, and ionic silk solution can produce silk fibroin films. Additionally, the fabrication of silk films using spin coating and the Langmuir–Blodgett (L.B.) technique are frequently adopted [68]. Very thin films have also been created using spin-assisted or manual layer-by-layer deposition processes [56]. Techniques including water annealing, controlled drying, alcohol immersion, and stretching are employed to increase the β-sheet crystallinity because the stability of such cast films is low [69]. For directed and accelerated cell growth or to alter the optical qualities, it is frequently important to manipulate the surface properties of silk films. To achieve such features, complex printing techniques and lithography are used [70].

### 4.3. Wet-Spun and Electro-Spun Fibers

Electro-spun nanofiber silk mats are advantageous for cell seeding due to their substantial surface area and porous nature [71]. Three-dimensional nanofiber constructions are utilized for blood vessel grafts and as nerve guides [72]. Regenerated silk fibers are also produced via wet spinning and micro-fluidic solution spinning. Wet-spun fibers, which may be generated on a far greater scale than nanofibers, typically have a diameter in the micrometer range [73]. The capacity to customize fiber structure and characteristics based on application and the assimilation of biomolecules when regenerated from silk solution are merits of such reinforced fibers over natural silk fibers [74].

### 4.4. Silk Hydrogels 

Silk hydrogels are created when aqueous silk fibroin solution goes through the sol–gel transition in the presence of dehydrating agents, acids, sonication, ions, and lyophilization [75]. The sol–gel conversion can be hastened by raising the temperature, and protein concentration and adding Ca^2+^ [76]. For non-injectable and injectable delivery arrangements, silk hydrogels can indeed be helpful. It has been discovered that the mechanical characteristics of silk hydrogels are excellent for creating scaffolds for load-bearing tissue engineering applications such as cartilage regeneration [77].

### 4.5. 3D Porous Silk Scaffolds

Freeze drying, porogen leaching, and solid free-form construction processes are used to manufacture silk scaffolds [78]. The pores in the freeze-dried sponges are smaller than 100 μm, and the pores’ diameters may be altered by varying the freezing temperature, the solution’s pH, and the number of organic solvents used [79]. Pore diameters can be increased from 60 to 250 μm by several cycles of freezing and thawing [80]. Using gas-foaming or solvent-casting leaching techniques can improve control over the pore structure. Porogen-leached 3D silk scaffolds are frequently employed in tissue engineering applications, primarily bone and cartilage, because of reasonable control over porosity and pore sizes [81]. Composite silk 3D scaffolds are designed to achieve good biological and mechanical performances by integrating organic or inorganic fillers [82]. The fillers are often introduced during the scaffold fabrication to guarantee their uniform distribution. However, it is also documented that particles can be integrated after production. The compatibility of the components presents a challenge in composite design. Phase separation, homogeneous mixtures, and unfavorable tissue reactions result from component incompatibility [83]. To raise the modulus of the scaffolds even further to approximately 13 MPa, reinforcing with fine silk strands is introduced [84]. Creating implantable tissue constructs without needing metal supports calls for improving the fabrication of biomaterials for bone repair. Scaffolds for ligaments are composed of silk composites reinforced with knitted silk mesh. It was discovered that there is homogenous cell dispersion throughout the construct 24 weeks following implantation. The results indicate the applicability of silk composites when mechanical qualities are crucial [85].

## 5. 3D Bioprinting in the Tissue Engineering Field

**Figure 1:**
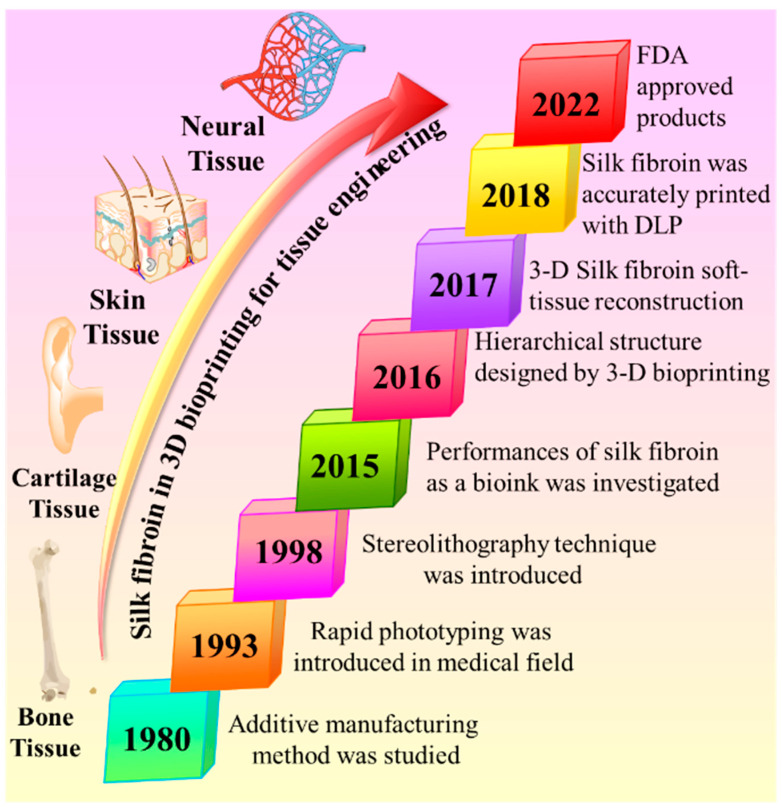
3D bioprinting has evidenced significant development and potential applications for the biomedical area.

For the past two decades, 3D bioprinting has been a convincible approach to the biomaterials area, and it is a way for a hopeful switchover from routine clinical therapies. Furthermore, several established 3D bioprinting approaches have been recognized to attain functional as well as structural reliability with the prototypical scheme, and it denotes that economically industrialized technology is ready-to-use for organ transplantation, implantation, and tissue regeneration [86,87,88]. The vital part of 3D bioprinting is bioink, an essential component for successful 3D products. Cell encapsulation, growth factors, drug delivery, and regeneration for medical applications are especially static in the developmental process of 3D bioprinting. Hence, finding a more suitable bioink for 3D bioprinting technology is crucial. Bioinks perform as cell-encapsulating materials and are used to construct the products in a 3D printing process. Therefore, it should be cell-friendly to 3D cell culture as well as the printing process [89]. In tissue engineering applications, cells, cell substitutes, and biomaterials are the three crucial components to healing damaged tissue. Hence, this, as well as hemocompatibility, cytocompatibility, cell encapsulation, and suitable mechanical strength in the biological environment are the essential pre-demandable characteristics of the material as a bioink. Silk fibroin (SF) proteins received more interest for biomedical applications, mainly focusing on tissue regeneration and repair [90,91,92,93], due to their extracellular matrix, cost-effectiveness, adjustable mechanical strength, controllable biodegradation, and hemo/biocompatibility [94,95]. Over the last three decades, the growth of SF-mediated bioink in 3D bioprinting has evidenced significant development and potential applications for the biomedical area, especially in tissue engineering, as shown in Figure 1. Therefore, the advantages mentioned above and developments were encouraged to promote the exploration of the SF as a bioink. Briefly, 3D bioprinting is an emerging technology transformation process from basic research to an advanced industrial revolution as it becomes commercially valid for precise, personalized medicine and organ or bone implantation [96,97,98,99,100,101,102]. 

## 6. 3D Bioprinting Technology for Silk Fibroin Bioinks

**Figure 2:**
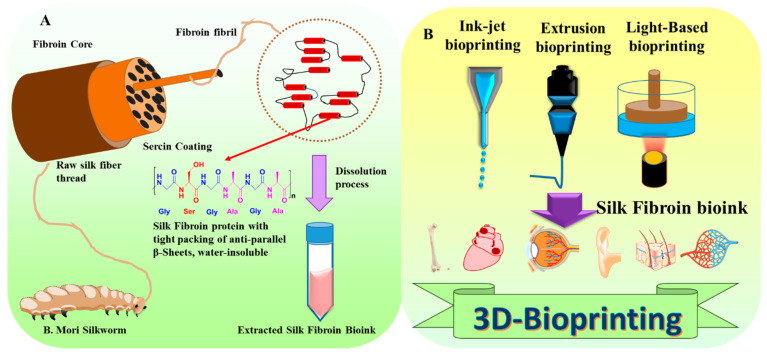
(**A**) Schematic representation of the SF-based 3D bioprinted scaffold from fundamental to biomedical applications. (**B**) Various types of 3D- printing techniques used for preparation SF-based 3D bioprinted scaffold in Tissue Engineering applications.

Briefly, 3D bioprinting is applied for fast design technologies to print cells, biomaterials, and growth factors in a layered form to generate biomimetic organ/tissue components. Further, 3D printing is a modern application of additive manufacturing techniques. The additive manufacture produces a compound of a 3D-biocompatible structure by depositing biomaterials on a substrate utilizing a computer-aided manufacturing method. Based on various molding basics and bioprinted scaffolds, bioprinting technologies are categorized into inkjet, extrusion, and photocuring-based bioprinting (Figure 2); the summary of general 3D bioprinting techniques is mentioned in Table 2. 

### 6.1. Inkjet Bioprinting 

In 1984, Charles Hull invited the first bioprinting machine from standard inkjet printers through the attachment of a mobile vertical axis stand, and the ink cartridge was converted into bioink tanks [113]. The ink cartridges released the bioink as droplets and slowly deposited them onto the printing stage through the stimulation of piezoelectric/thermal forces [114,115]. The bioink droplets can be placed in accurate 3D sites, resulting in a predefined structure. After the structure formation, stability was achieved through a different functionalization approach, resulting in shape preservation. These approaches provide distinct forms with minimal error and simple mechanisms [116]. The main limitation of this approach was that constructed materials showed poor mechanical strength without supporting a secondary process [117].

Moreover, cell loading becomes a challenging task in the 3D bioprinting process due to the presence of a diameter-sized inkjet nozzle and the obligation of poor viscosity, which creates thermal/mechanical traumas on the cells [117]. Hence, a limited number of bioinks are available for the inkjet bioprinting technique. Among them, SF is one such bioink, because of its low viscous nature, even upon a lack of modification. However, the secondary process was required to strengthen the materials and maintain the 3D shape. Recently, Rider et al. developed SF-based dental barrier membranes using inkjet bioprinting. In this investigation, deposited SF droplets were treated with methanol, leading to rapid stabilization through the β-sheet formation. However, this methanol-stabilized SF showed cytotoxicity and was unsuitable for cell encapsulations [118]. An investigation was recently reported to achieve a 3D structure, shape retention, and cell-sociable secondary process. In this study, horseradish peroxidase and sodium alginate were mixed with SF solution to form droplets. Later the developed droplets were treated with calcium chloride to stabilize the material through the ionic crosslinking of the alginate compositions.

Moreover, in the secondary process, the developed materials were treated with hydrogen peroxide, stimulating the horseradish peroxidase for the enzymatic crosslinking of tyrosine molecules in the SF The secondary process formed covalent bonds that could grip the constructed 3D structure and shape. This study showed that these dual-branched approaches have hopefully been utilized for NIH/3T3 cell-loaded scaffold bioprinting [119]. In another work, Suntivich et al. established this approach with polymer-grafted SF using polylysine and polyglutamic molecules, developing anionic and cationic regions on the protein that can enhance the stability through the electrostatic interaction of the polymers. This study showed that this approach had been applied for printing a layer of E. coli bacteria [120]. Although the most primitive form of 3D printing, the edition of SF into inkjet bioprinting was merely instigated in the last few years because the SF properties are more compatible with these printing techniques because of the challenges in accomplishing functional group crosslinks that aid cell survivability, and consequently, the minimum number of reports are presented on this technique. In addition, the small diameter nozzle size and the slow speed of printing subsequently decrease the cell survivability and reduce the adoption of SF-mediated bioink in inkjet technology. 

### 6.2. Extrusion Bioprinting

Extrusion bioprinting has received significant interest from various materials scientists because of its ease of design and availability for multiple bioinks [121,122,123]. The working principle was based on the predefined manner of bioink deposition as a continuous filament to form a 3D structure. The major challenge in using SF bioink in this technique is its poor viscous nature in diluted solutions and its hard-to-fix design after printing. Therefore, the improvement of bioink viscosity through various functionalization or additive agents is additionally fixed with the SF bioinks. For instance, Zheng et al. generated the β-sheet by adding low molecular weight polyethylene glycol 400 (PEG400). The synthetic biopolymer PEG400 stabilizes SF bioinks by inducing the β-sheet formation, and also, the bioink maintains its biosafety without affecting cellular viability.

Furthermore, a mixture of SF/PEG400 bioinks was printed with hMSCs and exhibited the multiplication of cells within the SF/PEG 3D structure for up to 15 days [124]. Similarly, Rodriguez et al. established novel freeform printing by introducing bioinks as a suspension in a PEG400 and laponite bath mixture. In this bath, the PEG400 aids in β-sheet formation for stabilization along with laponite for the improved suspension of bioinks. The laponite revealed non-cytotoxicity with the encapsulated cell hSMMs and showed more than 90% cell viability [124]. In another work, Jose et al. established the stability of SF bioinks by mixing glycerol and adonitol during the printing process and showed excellent cellular viability [99]. Of late, Chen et al. introduced a new approach to enhancing the strength of printed structure via a photochemical reaction. The studies showed that the surface of SF particles was altered with gelatin methacryoyl, increasing the particle size up to 900 nm and showing high viscosity in bioink, improving the printing resolution. The presence of methacrylate molecules in gelatin methacryoyl aids in generating the photo-crosslinking of the polymers through light absorption, further strengthening the printed final products. The final products encapsulated with NIH/3T3 and HUVECs exhibited 70% cell viability [125]. Sakai et al. investigated the SF nanofibers by incorporating different biopolymer solutions, including sodium-alginate, chitosan, gelatin, and hyaluronic acid. The investigation exhibited that the incorporated biopolymers enhance the SF bioink viscosity and reduce the widening of filaments during printing.

Moreover, the SF nanofibers and mixed polymers did not reduce cell viability [126]. The extrusion bioprinting of SF bioinks would commonly be meant for constructing products with higher mechanical strength. The several reported applications of this technique are for creating tissues frequently applied to more robust mechanical masses, for instance, bone and cartilage. However, this method’s primary limitation is its low accuracy in printing compared with other existing inkjet and light-based bioprinting. Reducing the width size of the nozzle improves the precision of bioprints, but it will indirectly raise the shear tension on cells and consequently reduce cell feasibility.

### 6.3. Light-Based Bioprinting 

In recent years, light-based bioprinting has been an emerging technique in the area of 3D bioprinting and received significant attention from materials science researchers worldwide because of its accuracy in the final printed structures [8]. Applying photo energy, photosensitive materials are crosslinked in the accurate site of the biomaterial region onto the bioprinted construct and encapsulate the cells and growth factors inside it, as is stimulated during the process. Applying peculiar light energy to produce photo-responsive materials motivates us to attain high-resolution bioprinting through this approach. Currently, there are two major classes of light-based printing: laser-induced forward transfer and digital light processing. The laser-mediated technique used single-pulsed radiation to stimulate the photosensitive crosslinking reaction in biomaterials, and then a single drop of the receiving substrate was placed [127]. On the other hand, the digital light processing method used ordinary light sources to stimulate photosensitive crosslinking between the biomaterials [128]. Two photosensitive reactions are used during this printing process, such as (i) the homogenous dispersion of the photoinitiators with SF bioink, which would then be crosslinked upon light irradiation. The crosslinked interpenetrating network contains SF chains with improved tensile strength. For instance, Lee et al. handled this approach through the addition of gelatin methacryoyl into SF bioink and applying photoinitiators in digital light processing. In this investigation, the printed biomaterials have an improved flexible modulus on increasing the amount of SF, and the encapsulated cells exhibited enhanced growth performances for 7 days [129]. (ii) The surface of SF was modified by grafting photosensitive molecules and adding a photoinitiator to generate covalent bonding in the biomaterials [128]. The reactive functional groups (primary anime and hydroxyl) on SF aid in grafting the photosensitive molecules onto the protein chain. For instance, Kim et al. introduced the glycidyl methacrylate onto the surface of SF to conjugate photosensitive methacrylate molecules and promote the photo-crosslinked covalent bonds between the biomaterials via an epoxide ring-opening reaction. In this study, the digital light process-based bioprinted scaffold holds NIH/3T3 and human septal chondrocyte cells, demonstrating cell viability for up to 14 days [102]. Similarly, Ajiteru et al. established a modified SF bioink with back-to-back glycidyl methacrylate and graphene oxide grafting. The addition of graphene oxide develops the electrical conductivity property in SF bioink for engineered neural tissue. The DLP-printed structure shows moderately strong mechanical behavior [130]. The light-based bioprinting technique can use photons to generate high-resolution 3D structures without affecting the encapsulated cells, which is the main advantage, whereas the usage of toxic photoinitiators and requiring an excess range of radiation in conjugation with bioprinting are the major demerits of this technique [131]. Commonly, the generation of free radicals from initiators in the polymerization process and the excess usage of radiation can roughly reduce the viability of the encapsulated cells [132].

## 7. Applications of 3D Bioprinting Based on Silk Fibroin

### 7.1. 3D Bone Tissue Engineering

Bone is a connective tissue that acts as a fundamental structure for the human system. Bone performs vital functions such as enabling body movement and safeguarding important organs from impairment [133]. It consists of a both organic and inorganic matrix, and the organic matter of the bone contains collagen [134,135]. Hydroxyapatite is the main component of the inorganic matrix of bone, and the rest is the combined form of salts and carbonates [136]. Hence, collagen and hydroxyapatite are the vital compositions of the bone and deliver strength and hierarchical structure to the bone [137]. The requirement of bone tissue engineering (BTE) arises in the case of oncological, congenital, and traumatic injuries that occur in bone and developing dangerous size flaws that bone fails to cure itself [138]. Hence, the designed biomaterials for applications in BTE should promise matrix strength and permit extracellular matrix deposition. The emerging 3D bioprinting techniques in the tissue engineering area offer a novel path to personalized bioprinted structures that comfort patient-specific organ/tissue damages [139]. SF-mediated bioinks have mainly been applied with extrusion printing techniques to achieve BTE because they are essential to strengthening the mechanical property during the printing process [140]. Most of the reported works in the literature denote that the SF-based bioinks for BTE commonly include SF-incorporated bioink composites with excellent viscosity before bioprinting. A secondary process is needed to enhance the mechanical characteristics of the deposited constructs.

In 2015, Das and co-workers first established the significance of SF bioink for BTE. The bioprinted scaffold holds gelatin-conjugated SF using crosslinking agents HRP and H_2_O_2_. The bioprinted structure embedded with human nasal inferior turbinate mesenchymal stem cells and its constructs can provide osteogenic expression once treated with the right osteogenic [141]. Chawla et al. demonstrated a new scaffold for tissue engineering with a similar SF bioink embedded with mesenchymal stem/stromal cells [142]. Recently, Wei et al. developed a novel SF bioink that incorporates hyaluronic acid, gelatin, and β-tricalcium phosphate, which is applied for platelet-rich plasma (PRP) therapy post-deposition. The bioprinted structure exhibited the PRP post-therapy-endorsed osteogenic expression in this investigation. These studies recommend that SF bioink can support embedded cells expressing osteogenic markers and allow the cells to form bone extracellular matrices later [143]. The solid mechanical property is vital for the constructed biomaterials in the application of BTE. In this regard, Ting et al. developed a 3D bioprinted SF structure with hydroxyapatite and sodium alginate, which exhibited great compressive strength and porosity. In addition, the 3D designed biomaterials increase proliferation and mesenchymal stem/stromal cells, which could interpenetrate into scaffold structures [144]. The designed bone scaffolds with a more excellent size range than (150–200 μm), restricted the oxygen diffusion inside the tissue. The absence of vascularization in bioprinted bone generally reduces the osteogenesis process and leads to bone implantation failure.

**Figure 3:**
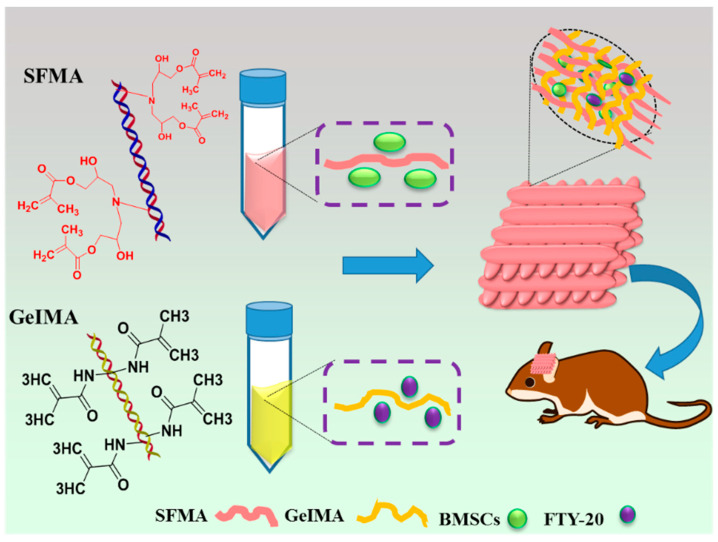
Schematic representation of the synthesis GeIMA/SFMA-based 3D printing scaffold embedded with BMSCs and FTY-720 for bone regeneration [145].

Regarding this issue, Yang et al. established SF-mediated bioink to integrate methacrylated gelatin (GeIMA) and methacrylayed SF (SFMA). They prepared the 3D bioprinted scaffold based on GeiMA/SFMA bioink with Bone marrow stromal cells (BMSCs) and fingolimod (FTY-720) drugs for the combined actions of osteogenesis and vascularization at the time of bone repair. The bioinks exhibited potential advantages in this study because of their adjustable rheology, rapid photo-crosslinking, and enhanced structure reliability following bioprinting to form BMSCs/FTY/G-S (Figure 3) [145]. This proposes that it is essential to integrate different bioprinting technologies to attain the required mechanical strength for bone tissue engineering. 

### 7.2. 3D Cartilage Tissue Engineering

Cartilage tissue is a specialized connective tissue that performs as a soft lubricated medium for articulation and aiding weight conduction through a low resistance. Though, it only has the minimum ability for essential curing capability because of a lack of blood, i.e., avascular necrosis [146]. Clinical curing approaches are critical to recovering the function of injured cartilage. Various clinical therapies have been applied to repair injured cartilage with minimum clinical success in attaining full recovery and reducing adverse responses [147]. Hence, the advancement of novel biomedical devices through tissue engineering approaches can support the delivery of full recovery from the above issues.

Moreover, most tissue engineering application investigations focus on cartilage tissue regeneration; the clinical transformation of the engineered cartilage tissues still requires a lot of time to fulfill [148]. To promise a 3D print of engineered tissue, the scaffold requires adequate mechanical power to maintain its printed structure and potentially oppose the heavy weights that native cartilage usually faces. SF-mediated bioinks have received significant attention due to their great mechanical capacity through crystallinity, supported by regulating the β-sheet quantity production [149]. Moreover, SF is considerable for 3D bioprinting engineered cartilage tissue structure because of its flexibility; it could be modified into various forms and maintain its hydration level [150]. The rate of degradation of the scaffolds can be customized, such as embedded chondrocytes can change the scaffolds along their extracellular matrix on time [151]. Hence, SF was an excellent bioink choice for engineered cartilage production. In 2016, Chameettachal et al. started the first investigation on a 3D printable SF scaffold for cartilage tissues. In this study, the SF and gelatin were enzymatically crosslinked with HRP and H_2_O_2_ to form a bioink with rheology adaptable for the extrusion printing technique. The bioprinted structure also helps to preserve cellular feasibility. In addition to this, the embedded cells expressed higher chondrogenic markers. This approach opened a new window to SF-based bioinks for cartilage tissue engineering [152].

Shi et al. designed the SF bioink with a mixture of silk and gelatin solutions in a ratio of 1:2 that could be applied to develop scaffolds with sturdy mechanical strength and degradation rates through 3D bioprinting for applications in cartilage regeneration. In vitro investigation revealed that the prepared scaffolds have chondrogenic differentiation capacity, and the innate chondrogenic cells were observed after 21 days. In vivo studies exhibited that bioprinted constructs embedded into defective rabbit cartilage tissues restored the cartilage deficiency in 4 months [153]. In another study, Kim and co-workers demonstrated an SF-mediated bioink through methacrylating SF and constructed a scaffold for cartilage regeneration utilizing digital light processing. In this study, the bioprinted structure showed an excellent compressive modulus (910 kPa) which denotes the ability to maintain 7 kg of kettlebell weight. In in vitro investigations, after four weeks of incubation, the scaffold showed efficient cartilage production and the occurrence of chondrocytes [102]. 

**Figure 4:**
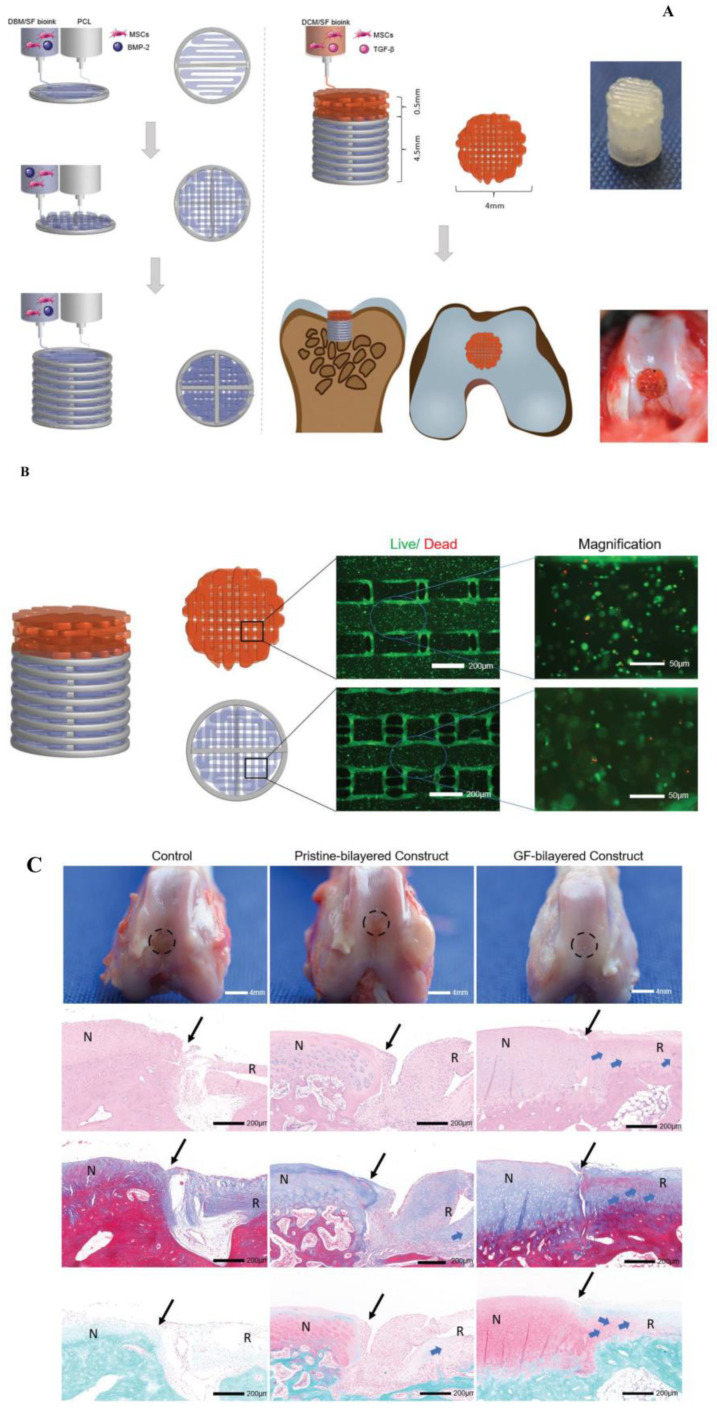
(**A**) Schematic representation of the preparation of the 3D bioprinted bilayered scaffold embedded with growth factors for osteochondral repair. (**B**) Cell viability of embedded BMSCs in the scaffold and fluorescence imaging of cartilage layer and bone layer incubated with BMSCs. (**C**) In vivo investigations of the bi-layered scaffold-assisted osteochondral tissue repair. Photographic image implantation studies of the control, pristine, and G.F. bilayered scaffold after 3 months. The Hematoxylin and Eosin staining of regenerated tissue after 12 weeks. Reproduced with permission [154]. Copyright 2021, Whioce Publishing.

Zhang et al. demonstrated a bioink containing SF and a decellularized extracellular matrix (dECM-SF) for two layers of the printed scaffold. Initially, polycaprolactone was printed as the first layer to form the bone-like frame, and later, dECM-SF solution was applied to develop the cartilage layer onto the frame. The bioprinted constructs imitate the native tissue by regulating the components, mechanical strength, and growth factor discharge on both layers of the scaffold. In vitro investigations of the scaffolds showed a moderate degradation rate and mechanical strength. Moreover, the scaffold performs as a controlled release system for the embedded growth factors (transforming growth factor-beta (TGF-β) and bone morphogenetic protein-2 (BMP-2) to promote cartilage repair. In vivo studies in the rabbit knee joint demonstrated that scaffold-embedded growth factors stimulated osteochondral regeneration [154] (Figure 4). In another work, Zhang et al. fabricated the crosslinker-free bioink with SF and decellularized the extracellular matrix and mixed it with BMSCs to generate the bioprinted constructs. The physically crosslinked scaffold loaded with BMSCs and TGF-β3 showed the capacity to stimulate chondrogenesis. Recently, Li et al. developed an HRP-crosslinked SF and tyramine-substituted gelatin 3D bioprinted hydrogel scaffold by extrusion printing. The hydrogel scaffold integrated with stem cell aggregates holds promising applications in cartilage tissue regeneration [155]. 

### 7.3. 3D Neural Tissue Engineering

The nervous system is a collection of specific cells arranged in a multifaceted network capable of combining and accepting signals from the physiological system. The nerves carry the sensorial response from the surroundings and pass signals to the cells/muscles to perform motor commands. Commonly, trauma or neurological disorder is the primary reason for neural damage; subsequently, it reaches life-long infirmity [156,157]. The lack of nerve regeneration ability of nerves after damage motivates the development of a synthetic nerve channel for solving the issue of nerve repair [158,159]. However, developing a viable nervous system with excellent characteristics is still tricky. The peripheral nervous system includes various components with a hierarchical structure. Each one has unique physicochemical properties that signal every cell’s activities. This understanding supports developing the nerve tissue scaffolds [160,161].

Moreover, endogenous electric fields have been exhibited to perform as behavioral signals during nerve reform. The bioprinting technique can effectively support the design of nerve scaffolds due to its power to bioprint well-organized constructs that permit neural cells to proliferate in a defined path. It also provides space for embedded cells in 3D surroundings that it requires to proliferate properly [162,163]. The primary factor in the neural construct is the necessity to include conducting property for developing electric conductance in the cells. This conductive property supports the cells to generate cell signaling and provides cell proliferation [164]. In addition, the conduction in bioinks would aid with breach-communication among the cells. The SF-mediated scaffold for engineered neural tissue largely relies on the structure’s conductance property and good mechanical strength. Zhao et al. demonstrated synthetic neural conductivity on SF-mediated bioink using 10% polyethylene oxide with silk and constructed the scaffold, following treating the construct with pyrrole solution to generate a surface coating of polypyrrole. The conducting property was established in the scaffold and later embedded the primary Schwann cells on a scaffold that could proliferate for five days. The spreading of the Schwann cells on the scaffolds was confirmed by staining the cells, showing positive for S100b markers [165].

**Figure 5:**
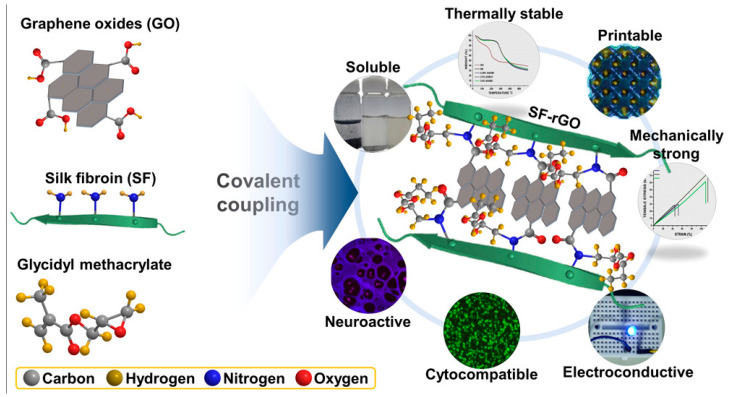
Schematic representation of the 3D bioprintable electroconductive bioink-mediated graphene oxide grafted SF Reproduced with permission [130]. Copyright 2020, ACS.

In another approach, Ajiteru et al. demonstrated the neural conductivity of a 3D bioprinted graphene oxide-grafted SF protein construct (Figure 5). In this study, the bioink was created with the dual grafting of graphene oxide and a methacrylate group on the silk protein, leading to bioprinting the scaffold using DLP deposition. The scaffold with embedded cells exhibited viability for 5 days and established the expression of the neurogenic markers (NeuN and α-tubulin) [130]. The 3D environment is essential for neural tissues to conduct proper signals between the cells; in this way, bioprinting plays a potential role in neural tissue engineering [163]. Silk-mediated bioinks can fulfill these needs by delivering an adaptable 3D scaffold using bioprinters with reasonable mechanical strength [166]. The grafting of various other molecules onto silk protein potentially develops and enhances the electrical conduction property in the scaffold [167]. These 3D neural scaffolds can be applied to model disease design and aid grafts in patients suffering from a nerve disorder. Recently, Sun et al. established that silk bioink could be modified to generate micropatterns on a peptide-doped substrate with the seeding of neuronal cells [168].

### 7.4. 3D Skin Tissue Engineering

**Figure 6:**
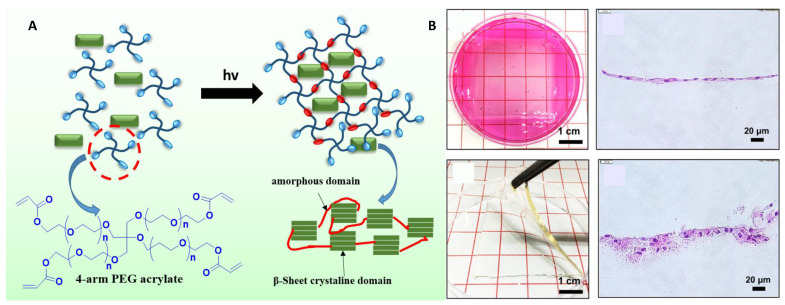
(**A**) Schematic representation of the synthesis of SF photo crosslinked 4-arm PEG scaffold [171]. (**B**) Photographic images of the formation of a 3D bioprinted scaffold of keratin layer with fibroblast cells. The hematoxylin and eosin-stained after the incubation for two and six weeks. Reproduced with permission [171]. Copyright 2019, Elsevier, Science direct.

The skin is the biggest multilayer compound organ in the physiological system, which consists of three layers (epidermis, dermis, and hypodermis) [169]. Commonly, two main approaches are handled to repair skin damage: autografts and allografts. However, these strategies are still limited in donors and recipients due to pain, dermal vascularization, and epidermis functionalization [170]. For the first time, Kwak et al. introduced the digital light process technique for designing a 3D bioprinted skin scaffold with SF and four-arm polyethylene glycol. The scaffold promotes cell proliferation and spreading, subsequently, the generation of a keratin layer (Figure 6) [171]. In another work, a gelatin-sulfonated silk-based layer of skin was developed through bioprinting with embedded growth factors; consequently, the scaffold exhibited skin-like tissues and improved skin regeneration through 3D bioprinting technology [172]. However, SF, as bioink to 3D bioprinting skin scaffolds, is starting, and the currently available data concerning the development of skin tissues and their biological characterization of the 3D bioprinted grafts showed a valid potential in skin regeneration and Table 3 summarised the silk fibroin based bioink for 3D printing applications in the tissue engineering.

## 8. Challenges in Translating Silk Fibroin into the Medical Market

The transformation of laboratory research to commercial products is not a simple process; various tedious stages need to be traversed before reaching the market. In the case of silk fibroin, every year, approximately more than 700 research reports can be regarded as topics in which silk fibroin has been developed as a biomaterial for biomedical applications. However, out of these many quality research reports, only a few works upgrade to clinical trials. These outcomes should be appreciable because it does not contemplate the several research works that have been reported in the area of tissue engineering and regeneration without any commercial value for biomedical applications. In academic research, particularly for in vivo biomedical applications, silk fibroin is probably utilized as one of the materials inside the biomedical device. This is a general method in tissue engineering in which each component consists of a scaffold function for a particular purpose. Commonly, several biomaterials such as chitosan, gelatin, and alginate exist readily as standardized biomedical products that can be stored and stable for long durations, whereas in the case of silk fibroin, only one medical product is commercially available, Silk Voice^®^, which consists of hyaluronic acid-coated silk fibroin particles alone. Moreover, the raw source of silk fibroin exists as an unstable water solution with a high cost and difficulty in storing for a long period if related to other polymers. This restricts the technology transfer of raw silk fibroin to biomedical devices, making the method of silk more complicated than other polymers. It should be noticed that commercially available silk fibroin powder is only applicable for cosmetic products and shows poor solubility in water due to its method of production (ball milling). The duration of stability was studied only in a few basic investigations on silk fibroin but hardly on silk fibroin scaffolds. Some reports mentioned that silk fibroin has been able to adjust its properties such as molecular weight [185,186], β-sheet structure [187,188], and crystalline [189]. Globally, the FDA developed a system to necessitate a premarket appraisal for biomedical tools and categorize them into four classes depending on their potential harm. Table 4 summarized their classes [56]. Commonly, all the advanced, modern, high-risk tools based on class III that do not have matching tools existing in the market involve completing the requirement of premarket approval (PMA—510(k) clearance). The PMA (class III) process takes a longer duration and requires heavy investment, which does not apply to academic institutions and small-scale industries. Moreover, a PMA (class III) requires a gold-standard method that still raises challenges in the case of natural silk fibroin-based biomedical devices. For these reasons, all the commercially available products created from silk protein were approved under the section of premarket notification (PMN) 510(k) clearance. Most silk fibroin biomedical tools have been cleared under PMN—510(k), which confirms the device has similar technical properties to earlier biomedical devices that are currently available for similar applications. The PMN—510(k) approval needs minimal capital and clinical trials; by revising the history of certain silk fibroin biomedical tools, particular facts could be increased. Overall, most of the reported research works on silk fibroin biomaterials reveal any proper standardization methods needed to transform them into biomedical devices. This might provide not precise results when going to pre-clinical trials, reducing the chances of a permitted biomedical device. 

## 9. Clinical Trials and Commercial Medical Products

Over the last two decades, more than 10,000 original research articles have been reported on the applications of silk fibroin in each area of tissue engineering. However, silk fibroin has been approved to generate only some biomedical devices currently utilized in medical practice. Moreover, certain devices fail in clinical practice for their corresponding applications, leading to the cancellation of clinical approval. The clinical trials for silk-based medical products were performed in the United States of America, which provides more information about how many silk-based biomaterials were transformed into clinical devices (www.ClinicalTrials.gov, Accessed Date: 5 December 2022). The major distinction between commercial products and the academic research that potentially attains a clinical performance is striking. Currently, new biomedical devices are reported in clinical trials for silk fibroin such as SilkVoice^®^, Derma Silk, EPIFIBROIN, and SERI^®^ Surgical Scaffold [56]. Three out of four devices were owned by Sofregen Medical Inc. Currently, SilkVoice (NCT04315415, NCT03790956) and SERI^®^ Surgical Scaffold are the two products that are commercially available, and others have just finished their clinical trials. It should be noted that even after the clearance of clinical trials, it will not confirm the successive approval for marketing the devices, hence it is very challenging to know when those devices will reach public usage. In recent years, the most applied silk fibroin biomedical product for clinical practice is the SERI^®^ Surgical Scaffold. This is the first scaffold that passed the clinical trial and was approved for commercial applications in 2008 and named as SeriScaffold (K080442) under Serica Technologies Inc. After 2016, Sofregen Medical Inc. bought the whole rights of the SERI^®^ surgical scaffold and developed the global commercialization of the first silk fibroin-based medical product. In 2018, the same company developed a second biomedical device completely based on silk fibroin called SilkVoice^®^ approved under PMN—510(k). It is an injectable fluid in a combination of silk and hyaluronic acid as a biomaterial for the growth of the vocal fold. Many silk fibroin-based medical products for tissue engineering have been clinically approved, and they should be reaching the market in the near future, though it is very difficult to confirm if a device that has even completed all developments will reach the market or not.

## 10. Future Perspectives

Target-specific tissue regeneration is essential for therapeutic purposes. The created tissue should progressively attach to the biological system to support the functionality of living systems. Silk-based designs offer simple control over the matrix shape, the rate of deterioration, and the conformal adhesion to subcutaneous tissues with low immunotoxicity and good biocompatibility. Recent improvements in our understanding of the structure and processing of silk create new possibilities for applying different types of silk in tissue regeneration. Silk-based scaffolds showed beneficial applications to construct hard tissues where low biodegradation and strong mechanical qualities are essential. The capacity to modify silk morphologies for tissue-specific requirements and a deeper understanding of biological properties and degradation products are prerequisites for the successful use of silk-based materials in tissue engineering. In this context, hybrid composites that incorporate 100% silk in various matrix topologies exhibit encouraging outcomes. Whereas much of the present literature on 3D printing with silk has been devoted to tissue engineering, intriguing research employing silk for other uses can inspire future printing technologies. By adding elements such as nanoparticles, enzymes, antibiotics, growth factors, or antibodies, silk inks can be tailored for particular needs. Silk has been printed using inkjet technology that has been doped with compounds for applications, including the detection of bacterial contamination and hosting. The future of personalized and regenerative medicine is in situ 3D printing. Despite being in the early stages of technological development, trials have been documented to create printing methods, inks, and crosslinking procedures suitable for in vivo applications. By enabling tissue/organ regeneration and developing the bioprinting of deformable sensors that can conform to their original tissue/organs during the bioprinting process and tissue deformation because of regular activity, this advancement brings us closer to personalized clinical applications. Several developments are still required to achieve an in situ model that can help the mechanical, cellular, vascular, and innervation needs of tissues while also delivering a user-friendly technology for surgeons, maintaining sterility, and being safe.

## 11. Conclusions

Silk fibroin has exceptional characteristics, including the capacity to print complex structures with variable mechanical strength, and biological modification as needed, without photochemical additives. These characteristics are influenced by the crosslinking ability and manufacturing process of silk fibroin. The various ways that silk fibroin gelates allow for multistep crosslinking treatments to tailor the final hydrogel’s characteristics, as is necessary for in situ printing in a surgical environment. However, there are still a lot of ink formulation and characterization improvements to be attained before in situ 3D printing can be considered a reliable approach. For instance, establishing sterilizing techniques appropriate for the clinical setting, studying the mechanisms behind in vivo degradation, and standardizing silk fibroin extraction procedures are all crucial variables to look at. In the combination of embedding biomolecules, such as growth factors, cytokines, or medicines, the flexibility of the silk fibroin scaffold can play a significant role. This could enhance the implantation success rate of bioprinted constructs by addressing regenerative responses. The use of extrusion-based 3D printing processes can restrict the viscosity of silk as the fidelity of the shape is maintained after printing. To imitate the intricacy of extracellular matrices in the human body, silk fibroin performs exceptional tasks that can be further increased through the addition of other biopolymers. For instance, gelatin or hyaluronic acid can enhance the bioresponsive activity of hydrogels, polyethylene glycol can increase the printability of silk, and silk nanofibers can also be combined with several biomaterials to achieve bioprintability and enhanced mechanical results. Aiming to access shape fidelity while evading the potential in vivo cytotoxic effects of photoinitiators when photopolymerization is used, various formulations of silk bioinks should be investigated, characterized, and standardized. Scaling up fabrication will lead to increased use in the clinic. Although it is unlikely that a single biomaterial will be able to replicate the intricacy of tissues and organs, silk fibroin’s adaptability and tunable properties can serve as the basis for inks to meet a variety of 3D in situ printing requirements.

## Figures and Tables

**Table 1:** Inventory of new patents based on silk fibroin for tissue engineering applications (data retrieved from Google Patents on 14 December 2022).

Patent No	Patent Title	Name of Inventor	Date of Publication	Main Features
CN-109667059-B	Method for preparing silk fibroin biological tissue engineering scaffold by solvent spraying	RogerZhang, YaopengLin, JiadongXie, Xiaofeng Zhang, Min Yu, Mingguang, Miao Lei	7 January 2022	SF-based scaffold with high porosity and specific surface area Applicable as blood vessels, urethra, and cartilage
CN-113926000-A	Preparation method of silk fibroin drug delivery tissue engineering scaffold	Chen Ying, Cui Xin, Wang Rong, Zhang Peipei	14 January 2022	SF scaffold prepared by a coaxial electro-spinning method Applicable as a drug delivery scaffold
WO-2022180565-A1	Silk fibroin and related use for 3d bioprinting	Alessandra Baldwin, Christian Andrea di Buduo, Pierre-Alexandre Laurent, Erik Gatenholm, Hector MartinezItedale Redwan-Namro, Volodymyr Kuzmenko	1 September 2022	SF scaffold-based 3D bioprinting Bio print the ex vivo models to generate the hematopoiesis
CN-109096501-B	Silk fibroin three-dimensional porous scaffold and preparation method thereof	Zhang Qiang, Cong Han, Yan Shuqin, Biography of You Ren, Li Xiufang, Luo Zuwei	1 November 2022	3D porous SF-based scaffold with excellent mechanical properties and strong water absorption
CN-115227871-A	Silk fibroin biomaterial ink and preparation method and application thereof	Chen Mingxue, Li Yangyang, Fang Yongcong, new Monday, Yang Dejin, Shao Hongyi, Zhang Songyang	25 October 2022	SF-based bioink with excellent shear thinning property Used for 3D bioprinting without the added extra compounds
AU-2017359330-B2	3D vascularized human ocular tissue for cell therapy and drug discovery	Kapil Bharti, Russell Louis Quinn, Min Jae Song	10 March 2022	Developing a 3D blood-retinal barrier consisting of the choroid and retinal pigment epithelial cells
CN-113651974-A	Preparation method of photoinduced silk fibroin/gelatin co-crosslinked hydrogel suitable for 3D printing	Huang Yiyi, Lu Lingling, Yao Juming, Li Yongqiang, Shao Jianzhong, Sun Guangdong, Pan Xiaopeng	16 November 2021	3D hydrogel based on SF crosslinked gelatin without the added extra chemical initiators
CN-113527709-A	Modified tussah silk protein and 3D printing ink based on modified tussah silk protein	Gou Ma Ling, Yellow Magnolia, Yang Xiong	22 October 2021	light-based polymerizable modified tussah silk protein for 3D scaffolds with excellent mechanical properties and biocompatibility
CN-112843337-A	Silk bionic bioink and preparation method and application thereof	Li Fengyu, Yao Yingkai, Tang Yongtao, Liu Jing, Guan Diqin	28 May 2021	SF-based bionic bioink simplifies the 3D bioprinting techniques by avoiding the process of heating, spraying an adhesive, and ultraviolet curing
CN-106267370-B	Silk fibroin/cellulose 3D printing ink	Zhang Yaopeng, Huang Li, Zhu Yufang, Shao Huili, Hu Xuechao	3 January 2020	Reported SF combined cellulose bioink exhibits high viscosity, and curing speed with the self-gelling duration is 0.5–3 min

**Table 2:** The summary of general 3D bioprinting techniques.

3D Bioprinting Techniques	Speed/Cost	Resolution/Viscosity	Vertical Printing Ability	Cell Viability/Density	Advantages	Disadvantages	Ref
Ink-jet	Fast/Economic	50 μm/10 m Pa s	Low	~95%; Low	Capable of bioprinting materials, poor viscosity	Lack of continuous flow	[103,104,105,106,107,108,109,110,111,112]
Extrusion	Slow/Budget friendly	100 μm/30 × 10^7^ m Pa s	Good	~89%; High	Capable to embed rich cell densities	Viscous liquids only	
Light-based	Moderate/Expensive	10 μm/300 m Pa s	Moderate	~85%; Medium	Both solid and liquid-phase biomaterials are used for deposition	Thermal damage due to laser irritation	

**Table 3:** Silk fibroin-based bioink for 3D bioprinting applications in tissue engineering.

Types of Crosslinking	Combination of Bioinks	3D Bioprinting	In Vitro Models	Cell Density	Compressive Modulus	Printing Mode	Ref
Photo crosslinking	SF-GMA	Bone TE	NIH/3T3	1 × 10^6^ mL^−1^	75–94 kPa	DLP	[173,167]
	SF-GelMA	TE	NIH/3T3	1.5 × 10^6^ mL^−1^	-	DLP	[168,171]
	SF-PEG4A	Skin TE	NIH/3T3	1.5 × 10^6^ mL^−1^	15.5 kPa	DLP	[102,169]
Enzymatic crosslinking	SF-CAM	Cartilage TE	rBM-MSCs	Seeding 65%		Extrusion	[170,174]
	SF-G	Cartilage TE	hMSCs	1.5 × 10^4^ mL^−1^	18 kPa	Extrusion	[152,165]
	SF	TE	hASCs	1.5 × 10^4^ mL^−1^	-	-	[175]
	SF/elastin	Intervertebral disc TE	hASCs	2 × 10^5^ mL^−1^	440 kPa	-	[176]
	3DG-SF-SO_3_	Skin TE	CFFs	1 × 10^6^ mL^−1^	-	-	[172]
	SF-G	Cartilages TE	BMSCs	-		Extrusion	[153]
Physical/Chemical crosslinking	SF-Collagen	Knee cartilage TE	BMSCs	2 × 10^7^ mL^−1^	-	Extrusion	[177]
	SF-Chitosan	Cartilages TE	BMSCs	2 × 10^7^ mL^−1^	-	Extrusion	[178]
	SF-Alginate	Vascular tissue engineering	NIH/3T3	1 × 10^6^ mL^−1^	6.6 kPa	Inkjet	[119]
	SF-PEG	Cartilages TE	hMSCs	2.5 × 10^6^ mL^−1^	-	-	[179]
	SF-PEG	Cartilages TE	hMSCs	2 ×10^6^ mL^−1^	258 kPa	Stereolithography	[124]
	SF- collagen	Nerve TE	-	-	-	-	[180]
	silk-gelatin	Cartilages TE	Chondrocytes	-	0.1 mPa	Extrusion	[181]
	SF-PEG	Cartilages TE	PRP	-	110 kPa	-	[182]
Ionic crosslinking	SF-Alginate	Cartilages TE	NIH 3T3	1.5 × 10^6^ mL^−1^	-	Inkjet	[183]
	SF-G	Bone TE	hMSCs	2 × 10^6^ mL^−1^	-	-	[184]

**Table 4:** FDA classification of the devices and the path taken for their approval [56].

Types of Classes	Details	Applications	US Regulator Path
Class I	Minimal harm to the patient	Endoscopic instruments	5% 510 k (or) PMN approval
Class II (a) and (b)	(a) Moderate harm with limited period of device usage; (b) moderate harm only	Catheters, Ear-hearing tools,	510 k (or) PMN approval with clinical proofs
Class III	It has 10% substantial risks	Organ implants	Approval in some special cases only

## Data Availability

Not applicable.
